# Network features suggest new hepatocellular carcinoma treatment strategies

**DOI:** 10.1186/s12918-014-0088-0

**Published:** 2014-07-29

**Authors:** Orit Lavi, Jeff Skinner, Michael M Gottesman

**Affiliations:** 1 Laboratory of Cell Biology, Center for Cancer Research, National Cancer Institute, National Institutes of Health, 37 Convent Dr., Room 2108, Bethesda 20892, MD, USA; 2 Laboratory of Immunogenetics (LIG), National Institute of Allergy and Infectious Disease, National Institutes of Health, Rockville, MD, USA

**Keywords:** Co-expression network, Pathway network, Robustness, Redundancy, Resistance, Cancer therapy

## Abstract

**Background:**

Resistance to therapy remains a major cause of the failure of cancer treatment. A major challenge in cancer therapy is to design treatment strategies that circumvent the higher-level homeostatic functions of the robust cellular network that occurs in resistant cells. There is a lack of understanding of mechanisms responsible for the development of cancer and the basis of therapy-resistance mechanisms. Cellular signaling networks have an underlying architecture guided by universal principles. A robust system, such as cancer, has the fundamental ability to survive toxic anticancer drug treatments or a stressful environment mainly due to its mechanisms of *redundancy*. Consequently, inhibition of a single component/pathway would probably not constitute a successful cancer therapy.

**Results:**

We developed a computational method to study the mechanisms of *redundancy* and to predict communications among the various pathways based on network theory, using data from gene expression profiles of hepatocellular carcinoma (HCC) of patients with poor and better prognosis cancers. Our results clearly indicate that immune system pathways tightly regulate most cancer pathways, and when those pathways are targeted by drugs, the network connectivity is dramatically changed. We examined the main HCC targeted treatments that are currently being evaluated in clinical trials. One prediction of our study is that Sorafenib combined with immune system treatments will be a more effective combination strategy than Sorafenib combined with any other targeted drugs.

**Conclusions:**

We developed a computational framework to analyze gene expression data from HCC tumors with varying degrees of responsiveness and non-tumor samples, based on both *Gene* and *Pathway Co-expression Networks*. Our hypothesis is that redundancy is one of the major causes of *drug resistance*, and can be described as a function of the network structure and its properties. From this perspective, we believe that integration of the redundant variables could lead to the development of promising new methodologies to selectively identify and target the most significant resistance mechanisms of HCC. We describe three mechanisms of redundancy based on their levels of generalization and study the possible impact of those redundancy mechanisms on HCC treatments.

## Background

Hepatocellular carcinoma (HCC) is one of the deadliest cancers worldwide [1],[2]. Treatments of advanced disease are largely ineffective, mainly due to the lack of understanding of mechanisms responsible for the development of the cancer and the basis of therapy-resistance mechanisms. Cellular signaling networks have an underlying architecture guided by universal principles. One such principle is that networks include redundant variables. A robust system, such as cancer, has the fundamental ability to survive toxic anticancer drug treatments or a stressful environment mainly due to its mechanisms of redundancy. *Redundancy* means that two or more elements potentially could perform the same function and that inactivation of one of these elements has no significant effect on the biological phenotype or on the dynamic process. Consequently, inhibition of a single component or even an entire pathway would probably not constitute a successful cancer therapy. Choosing drugs for therapy is a complex task. Researchers often choose a specific element to target (e.g., the VEGFR2 tyrosine kinase inhibitor) using statistical analysis of gene expression, or the target’s ability to affect cell fate (i.e., does the target act as an upstream hub?). However, many cancer drugs fail or underperform due to redundancies in their target’s pathways or the existence of alternative pathways. Efficiently targeting pathways is problematic, because it is unclear whether we should identify pathway targets by level of expression or by their location in the pathway (e.g., upstream elements). There are many examples indicating that redundancy serves as a resistance mechanism with clinical implications. For instance, several different ABC transporters can confer resistance to the same drugs, so inhibitors must target all of these transporters to be effective in reversing transporter-related multidrug resistance [3].

In general, there are many computational and mathematical approaches to directly/indirectly address redundancy, such as network theory. Network theory helps to describe relationships between every pair of elements, where the elements could be genes, proteins, metabolites, etc. The relationships could be physical integration, correlations, targets, etc. The network could be as complex as we need it to be. For example, a multilayer network can include many components and types of relationships, depending on the objective [4]. Describing a system via a network can help to find properties that could potentially lead to treatment strategies, or better understanding the process. Redundancy in a network, for instance, can be expressed by the redundant paths that start at one node and end at another, or by the redundant nodes that are part of one layer and connect to the same node in the second layer. The main objective of this current paper is to describe the redundancy of biological function (i.e., pathway) that is modeled using the network framework and gene expression data.

HCC is known to be a heterogeneous disease. Thus, numerous genomic-based classifications have been proposed to describe its various forms. These kinds of studies indicate the complexity in finding a consistent molecular classification for such a problem. Gene expression profiling has been used extensively in cancer research, providing useful information. A prediction of patient therapeutic response based on tumor gene singularities would improve overall efficacy of molecular therapies used to combat HCC. Computational algorithms that predict the recurrence of HCC based on clinical, pathological, and gene expression data are the current approach in the field [5]. The studies by Hoshida and colleagues based on gene expression profiles highlight the significance of integrating multiple data sets to provide a robust molecular classification of HCC. They presented a meta-analysis of 9 independent cohorts, including 603 patients [6],[7], and defined three robust HCC subclasses (termed S_1_, S_2_, and S_3_), that were correlated with clinical parameters. The S_1_-signature reflected abnormal activation of the WNT signaling pathway, the S_2_-signature was described by the proliferation pathway as well as MYC and AKT activations, and the S_3_-signature was associated with hepatocyte differentiation. These three signatures were shown to predict the recurrence of HCC. S_1_ and S_2_ signatures had poor overall survival and those with the S_3_-signature had good overall survival.

However, gene expression profiling provides an incomplete picture, since it does not include communications among the genes. It is increasingly believed that cancer cells involve a large number of biochemical components that interact through complex networks and as a result, display nonlinear dynamics [4]. Therefore, a system level approach, rather than a gene-signature approach, is more appropriate to handle this level of complexity and will undoubtedly provide new insights for cancer research. Constructing a co-expression network is the next logical step following gene expression profiling. Gene Co-expression Networks (GCNs) have become a rapidly developing area of study with implications in cancer research [8]–[10]. A GCN is an undirected graph, with genes forming the network nodes, and significant relationships serving as indirect network edges [11],[12]. These relationships are usually defined as statistical correlations (e.g., Pearson, Spearman). A GCN does not necessarily include physical gene interactions as would be found in a genetic interaction network, but includes information on the gene connectivity with the entire system, which is usually overlooked in other types of statistical analysis [13]. The expression edges could be defined using other theoretical approaches [9],[14], such as using a generalized definition of the pairwise correlation, as in the mutual information method. One application of a co-expression network that poses computational challenges is the identification of functional gene modules (i.e., clusters of highly interconnected genes). One example of a module could be a signaling pathway [8],[15].

The problem of redundancy at the functional level has mainly been addressed by identifying differentially expressed pathways based on gene expression data by calculating activity levels for each pathway within the samples [16],[17]. The next development in this field was the quantification of relationships between co-expression pathways [18]. A pathway is not an isolated process. Most, if not all, signaling pathway activities are driven by crosstalk between other pathways within the same cellular network. Determining the design principles behind this network complexity is key to understanding the cellular activity. Crosstalk between pathways has an important effect on the dynamics of a system. For instance, it was demonstrated that pathway crosstalk can generate robust oscillations in calcium and cAMP concentrations [19]. Moving from specific crosstalk between two pathways to crosstalk between all pairs of pathways, researchers proposed a global pathway crosstalk network [20],[21]. This approach assumes that a pathway edge exists if significantly more protein-protein (P-P) interactions are detected between a pair of pathways than expected by chance. It is important to note that in individuals with cancer, the genetic and epigenetic changes that accompany malignant transformation alter the known P-P network, and thus a better reference network needs to be described.

**Figure 1:**
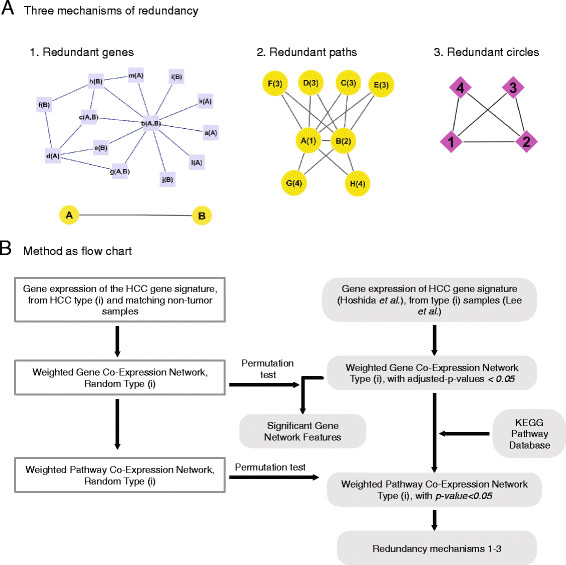
**Mechanisms of redundancy. (A)** All genes function as parts of pathways (e.g. A-H, shown as yellow spheres). Some genes (e.g., *a-m,* shown as purple squares) affect one another and form a gene sub-network (nodes are genes, and edges, shown as blue lines, describe correlations,). Theoretically, if we assume only two pathways **(A and B)**, this gene sub-network can be simplified to a single edge/crosstalk between the two pathways (gray lines). The perturbation of a single gene may not significantly affect the expression of this pathway crosstalk. Thus, the redundant genes in each pathway and their connectivity may act as a resistance mechanism. Moreover, each instance of crosstalk may be regulated by another pathway and form a simple network structure, such as *triangle*/*3-node circle*. If the network is well connected, a single crosstalk may be included in many 3-node circles (i.e., redundant paths per crosstalk). An indirect approach to perturb a crosstalk would be through its shortest paths. Having many alternative paths may lead to formation of a robust structure. Lastly, those alternative 3-node circles may be grouped together into categories (e.g., 1–4 in magenta diamonds) based on their biological system (e.g., pathways G and H share the same category number 4). Thus, instead of having six 3-node circles, we have two main category circles: [1]–[3] and [1],[2],[4]. These three mechanisms of redundancy reveal the impact of network structures on the level of resistance. **(B)** Our method of determining the pathway co-expression network is described here in steps as a flow chart (see Methods section for more details).

Our hypothesis is that redundancy is one of the major causes of drug resistance, and can be described as a function of the network structure and its properties. Therefore, methods that deal with this structural network problem should be developed. By this view, integration of all the redundant variables could lead to the development of promising new methodologies to selectively identify and target the most significant resistance mechanisms in HCC. Here, we develop a computational methodology to analyze gene expression data from HCC cells with varying degrees of responsiveness as well as non-tumor samples, based on both the Gene Co-expression Network (GCN) and Pathway Co-expression Network (PCN), where the reference network is constructed based on random sample selection from the two groups. We offered three mechanisms of redundancy based on their levels of generalization: *Redundant Genes* of a given pathway, *Redundant Crosstalk Paths* between pair of pathways, and the *Redundant Circles* (also known as triangles) of a given set of pathway categories (Figure 1A). Using the Hoshida *et al*. profiles [7], and the responsiveness classification of Lee *et al.*[12], we study the possible impact of those redundancy mechanisms on HCC treatments (Figure 1B). We find unique PCNs for better and poor overall survival. Our results reveal the distinctive effect of Immune System genes on HCC pathway crosstalk compared to the Signal Transduction genes that are mostly being targeted by current HCC treatments. This model can provide guidelines for better treatments that circumvent the resistance of HCC. Our system-level analysis reveals a possible reason for the limited effectiveness of current treatment strategies and demonstrates how treatment efficacy could be improved based on network connectivity.

## Results

In this section, we introduce three redundancy mechanisms based on network features, which include the two levels of gene and pathway analysis of both poor survival and better survival phenotypes. Figure 1A illustrates the 3 redundancy mechanisms (involving Genes, Paths, Circles), and a schematic diagram of the Pathway Co-expression Network method can be found in Figure 1B. Using the gene expression profiles of robust molecular classifications of HCC published by Hoshida and colleagues, we determined the initial differentially expressed genes [7].

Co-expression networks were constructed using clinical samples of 91 HCC tissues and 60 matched non-tumor surrounding liver tissues (containing both hepatitis B (HBV) and hepatitis C virus (HCV)). These samples were previously classified by Lee *et al*. into two groups of cancer based on overall survival, where group A demonstrated poor overall survival and group B demonstrated good overall survival [22]. The Gene Co-expression Network that was calculated in this study is an undirected graph, with genes forming the network nodes, and significant relationships, defined as Pearson correlations, serving as indirect network edges. Our new method of Pathway Co-expression Network is also introduced here. Only significant results are reported, and for more details see the Methods section.

We created a multilayered network that includes genes, pathways, and pathway families. We begin by examining the first redundant mechanism, i.e., the effect of *Redundant Genes* of a given pathway on the Pathway Co-expression Network connectivity (Figure 1A_1_). We then explore the abnormal crosstalk among the different genes and pathways that is significantly different from non-specific cancer networks. We address the important concepts of network theory to elucidate HCC resistance mechanisms (involving hubs, circles, network structure, and other properties) [4]. In addition, we present two other redundant mechanisms, *Redundant Paths* between pair of pathways (Figure 1A_2_), and the *Redundant Circles* of a given pathway category (Figure 1A_3_). We explore the biological implications of several pathway examples, and ask about their dependence on the gene level analysis. Lastly, based on the Type A HCC network, we estimate the impact of drugs that are currently under evaluation in order to optimize treatment.

### Genomic signatures of HCC poor and better survival phenotypes

The HCC poor survival phenotype signature proposed by Hoshida and colleagues [7] included 354 different genes with 169 unique pathways, while only 261 genes with 177 pathways were included in the better survival phenotype. Note that the number of genes per pathway is not uniformly distributed (Additional file 1). Sorting the top 10 pathways with their subcategorized descriptions, we observed that, prior to our analysis, in the poor prognosis phenotype most pathways belong to the subcategory *Cellular Processes,* while in the better outcome phenotype most pathways belong to the subcategory *Metabolism*. We apply these expression profiles and the computed Pearson correlations between every pair of genes, we study the Gene Co-expression Network and Pathway Co-expression Network of non-tumor samples (termed N) and HCC samples with poor (termed type A) and better (termed type B) survival groups using data from Thorgeirsson and colleagues [22]. Our goal was to provide a novel method of analysis that accounts for redundancy with a pathway-network perspective that highlights potential drug targets (for model details see Methods).

### Redundancy: limited effects of targeting a single gene on the entire network

Mutations in Mitogen-Activated Protein Kinase (MAPK) pathways are a frequent cause of increased cell proliferation, resistance to apoptosis, and resistance to other therapies [23]. There are currently many clinical trials evaluating MAPK pathway targeting in cancer patients, using inhibitors such as Sorafenib, Sunitinib, or Gefitinib, where the strategy is to target a gene or several genes, and thus affect the entire pathway. We show here that in the case of a well communicated pathway such as MAPK, this approach by itself may not yield promising results, as there are many redundant genes as well as redundant crosstalk involving other pathways.

**Figure 2:**
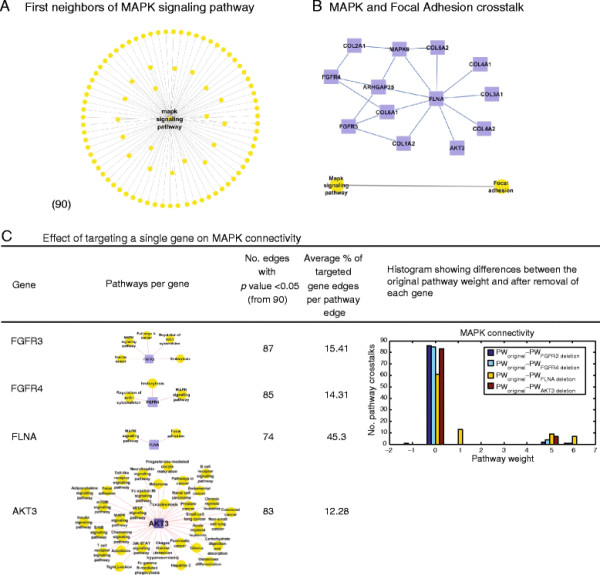
**Targeting the MAPK signaling pathway. (A)** The MAPK pathway communicates with 90 other pathways from the cancer type A network, including crosstalk with Focal Adhesion. **(B)** MAPK and Focal Adhesion crosstalk is basically the outcome of the sub-network of all correlated genes included in at least one of these two pathways. **(C)** Although all of these genes function as part of the MAPK pathway, they may have different effects on MAPK connectivity, since each gene correlates with different genes and pathways. Measuring the impact of each gene by the number of the resulting significant edges could give us a way to predict the gene’s involvement and intensity related to MAPK connectivity. For instance, FGFR3 is included in 5 pathways and on average is connected with 15% genes per pathway edge. The deletion of FGFR3 changes the number of pathways that crosstalk with the MAPK pathway, from the initial 90 crosstalks to 87 crosstalks.

We explore here one level of redundancy and study the changes in crosstalk between pairs of pathways when a single gene is targeted. We generalize the gene network to a pathway network, where each gene edge is translated into pairs of pathways. The pathway network is composed of pathways as nodes, and the weight of a pathway edge is the mean of all the gene correlations that form it. We use permutation re-sampling of the original data to model the null distribution and calculate the *p*-value of each pathway edge (see Methods). We demonstrate how targeting a specific gene of the MAPK pathway does not necessarily target its pathway and its communications with other pathways in the network (Figure 2). The MAPK pathway communicates with 90 other pathways with different degrees of intensity (weights) based on the poor responders network (cancer type A, Figure 2A). A gene sub-network can be simplified and translated into a single weighted pathway edge, or many pathway edges, depending on the initial information concerning gene and pathway relationships, differentially expressed genes in given cancer samples, and if the pathway edges are significantly different from a non-specific cancer case (Figure 2B). For example, four genes function as part of the MAPK pathway: FGFR3, FGFR4, FLNA, and AKT3 (these genes function as part of 5,3,2, and 35 pathways respectively). We estimate the effect of deleting each gene separately on the weight and *p*-value of the MAPK sub-network (Figure 2C). We found that the gene connectivity in a gene network and the number of pathways in which a gene initially functions are valuable parameters to estimate its effect on the pathway network, although they may have a very limited effect on the global pathway network. Our results demonstrate that in a case where a single gene with only two linked pathways is deleted, but that gene is correlated with many genes, the connectivity of the MAPK pathway sub-network is affected. For example, removing the FLNA gene reduces the connectivity of the MAPK pathway: from 90 to 74 pathways. On the other hand, the number of linked pathways is important when the number of correlated genes is low. Deletion of AKT3, which functions as part of 35 known pathways, (i.e., a central gene), reduces the size of the pathway sub-network to 83 pathways. This first redundancy-based mechanism shows the limited changes in the overall pathway crosstalk based on a single target gene, which may explain the occurrence of resistance to targeted cancer therapy.

### Simplifying the complexity: a systems approach to studying pathway network connectivity

As mentioned earlier, studying the structure of a network is a critical first step to reveal redundancy and resistance mechanisms. Here we explore several network properties that quantify the topology and complexity of both gene and pathway cancer networks (Additional files 2, 3, 4 and 5) and present our approach to finding targets based on network features. A weighted gene network based on Pearson correlations (with no threshold) and its higher-correlated sub-network (|correlation| ≥ 0.5) are constructed separately for each HCC type. Both cancer gene networks are well-connected, with many *circles* composed of 3 or 4 correlated genes. These special circle structures, which are composed of a limited number of genes that affect one another, have a direct effect on the probability of having modules and limit the effect of targeted therapy by offering redundant regulator motifs and/or feedback loops [24]. In both cancer gene networks there are more positive correlations than negative. In cancer type A, about 40% of the gene correlations are above 0.5, while in cancer type B only 17% are above 0.5 (in absolute values). To confirm phenotype specificity, we compared the properties of each network to the random networks, and found significant differences (see Additional files 2 and 3). Overall, both cancer gene networks have a structure that is less connected than random cases, with lower numbers of edges, edge densities, averages of node degrees, and averages of clustering coefficients, but with a higher number of nodes (network features are given in Additional file 4).

### Local regulations

**Figure 3:**
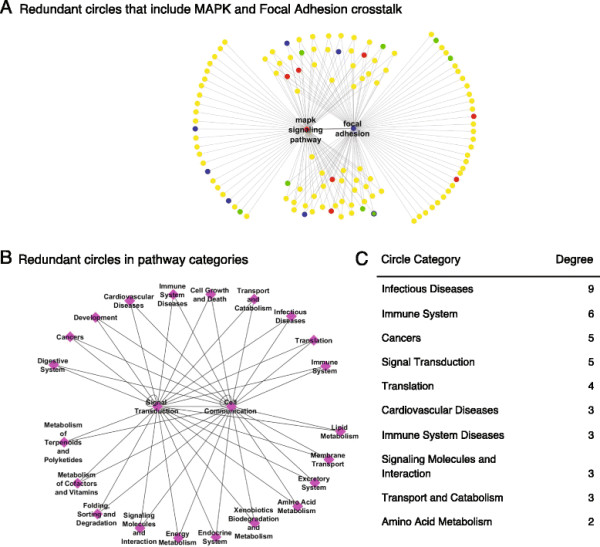
**Redundant paths between the crosstalk of MAPK and focal adhesion pathways. (A)** The sub-network of pathways that engage in crosstalk with either the MAPK pathway or the Focal Adhesion pathway are shown in yellow. Among them, pathways that are targeted by the drug sorafenib are red, pathways with the highest node degree (the first 8 hubs, see list in Figure 4A) are blue, and the immune system pathways (with highest node degree, see list in Figure 4A) are green. The 63 redundant 3-node circles included in this crosstalk highlight the complexity of targeting this crosstalk with an indirect approach, but on the other hand indicate its importance due to the many regulators, among them being the hubs, the immune system, and pathways targeted by sorafenib. **(B)** The redundant 3-node circles can be grouped based on their pathway categories (category shown in magenta), so a more compact meaningful representation of those circles can be written: instead of 63 pathway circles, we have 23 category circles. For example, 9 of the 3-node circles are from the Infectious Diseases category, and 6 others are from the Immune System category. **(C)** All pathway categories with degree above 1.

When the gene network is generalized to a pathway network, the structures of both cancer pathway networks (types A and B) appear to include many small circles (order of 3 and 4) that are connected to most pathways. For example, the crosstalk between the MAPK pathway and Focal Adhesion in the cancer type A network is included in 63 different 3-node circles (Figure 3). This is the second redundancy mechanism, shown as Figure 1A_2_. The 63 shared neighboring pathways can be grouped into 23 pathway categories. For example, two circles that are associated with the Lipid Metabolism category would be [MAPK pathway, Focal Adhesion, Steroid Hormone Biosynthesis] and [MAPK pathway, Focal Adhesion, Sphingolipid Metabolism]. Thus, this structure reveals multiple redundant circles from the same category combinations. Although there are thousands of small circles (network features are given in Additional file 5), most of them function as part of the same pathway categories. We investigated the pathway types of all pathways that are included in 3-node circles and found that for the cancer type A network (with high gene correlation), most of the pathways from the categories Immune System, Infectious Diseases, and Immune System Diseases are part of those regulated structures (Additional file 6). The most common type of 3-node circles combines three pathways from those three categories, i.e., 3 pathways with one from each category. The top 100 most common types include at least one of those 3 pathways (Additional file 6). As for the cancer type B network (with high gene correlation), most of the pathways from the categories of Amino Acid Metabolism, Carbohydrate Metabolism and Lipid Metabolism are part of those regulated structures. The most observed type of 3-node circles is the combination of [Amino Acid Metabolism, Carbohydrate Metabolism, Lipid Metabolism]. The categorized circles are the third redundancy mechanism, shown as Figure 1A_3_.

### The gap between gene hub and pathway hub

Another important feature of the network is *Node degree*. Node degree is the number of nodes/elements in a network that correlate/crosstalk with a specific node. The nodes with the highest connectivity values are referred to as hubs and are crucial to the entire system. We explore here several hubs from both gene and pathway networks (written as hub_gene_, hub_pathway_), of each cancer type and discuss their HCC clinical and experimental relevance and mention current progress in the field (see also Discussion and conclusions section). Gene and pathway hubs of both cancer types are listed in Additional file 7. In the poor survival gene network (cancer type A), the 10 top connected genes are LAPTM5, ASAH1, IQGAP1, GLIPR1, CD47, ARHGDIB, SRGN, RAB31, FCGR2a, and CD53. These 10 hubs_gene_ form a complete graph, i.e., every pair of genes is correlated by a unique edge (with |Pearson correlation| > 0.5), which demonstrates the global structure of this gene network, and may imply the existence of common regulatory mechanisms.

In the gene network of cancer type B, the 10 top connected genes are ALAS1, ALDH3A2, CTH, ACSL1, SRD5A1, HSD17B4, GLYAT, CBR1, HGD, and HADH. All of these genes are included in part in the metabolism pathway category: Amino Acid Metabolism pathways, 5 × Carbohydrate Metabolism, 5 × Lipid Metabolism, 1 × Energy Metabolism. Some of these genes share pathways. For example, the genes ALAS1 and CTH function in Glycine, Serine and Threonine Metabolism (Additional files 1 and 7). Recently, many metabolomics profiling of HCC have been reported, in which the above-mentioned pathways are confirmed in part by their independent metabolomics analysis findings, with additional important information on their communications [25]–[27]. In addition, the hubs_gene_ that function as part of metabolism pathways, also correlate with the Endocrine System and Transport and Catabolism pathways. One example is the hub_gene_ ACSL1, which is known to be involved in four pathways: fatty acid metabolism, PPAR signaling pathway, peroxisome, and adipocytokine signaling pathway. The PPAR signaling pathway is also a hub_pathway_ in the cancer type B pathway network. This hub highly positively crosstalks with 133 other pathways, among them being ABC Transporters, Adherens, Tight, and Gap Junctions. It also communicates with many HCC targeted pathways, such as Wnt, Tgf-beta, Vegf, Jak-Stat, Insulin signaling pathways, and is negatively correlated with Lysosome, RNA degradation, and Toll-like receptor signaling pathways. Interestingly, the opposite is true in the cancer type A pathway network, where positive crosstalk is observed between the PPAR signaling pathway and the Lysosome pathway, in addition to other positive crosstalk with pathways such as Cell cycle, Regulation of actin cytoskeleton, and Cytokine-cytokine receptor interaction.

Furthermore we asked, given the connectivity of each gene in the gene network, and the pathway information that initially was obtained (e.g., from the KEGG database), if it would be sufficient to assume that the most connected pathways (i.e., hub_pathway_) in the pathway network are nothing more than the pathways of hubs_gene_ or/and pathways that initially include many genes? Are there any hubs_pathway_ where this is not true? It appears that there are several hubs_pathway_ from the Cancer type A group that do not have these two characteristics. For instance, in the case of cancer type A, there are at least 5 pathways that are less connected than expected based on the two characteristics: Focal Adhesion, Lysosome, Gap junction, Sphingolipid metabolism, and mTOR pathway. Also, there are at least 3 pathways that are more connected than expected: MAPK, Wnt, and Jak-STAT signaling pathways. While in the case of cancer type B, most of the hubs_pathway_ have these two characteristics, except Glycolysis/Gluconeogenesis, which is less connected than expected.

### Current targeted treatments of HCC and key sub-networks of pathways

**Figure 4:**
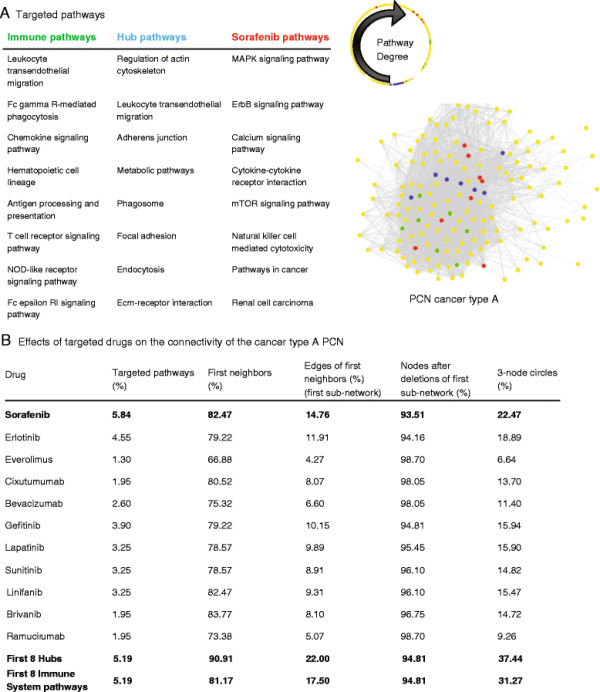
**Targeting strategies for cancer type A. (A)** The drug sorafenib targets 8 pathways in our network (red). We compared the network connectivity of the targeted pathways of each drug with the first 8 hubs (blue), and the highest connected Immune System pathways (green), with results shown in panel **B**. The targeted pathways have different node degrees, plotted as spheres, where the blue are the highest. All pathways that are not part of panel **A** table are shown in yellow. On average, the immune pathways are more connected than the pathways targeted by sorafenib. At right, illustration of the pathway network of cancer type A, with |gene correlation| ≥ 0.5. The compact structure of the network can be seen clearly by the dense area in the center, where all the immune system pathways are located. **(B)** The connectivity of the targeted pathways is measured by the first neighbors, the sub-network of the first neighbors, the number of nodes after deletion of the targeted pathways, and the number of 2-node circles of at least one of the targeted pathways. A list of each drug and its matching targeted pathways is given in Additional file 8.

There are currently several clinical trials evaluating targeted therapies for HCC, including Sorafenib, Sunitinib, Gefitinib, Lapatinib, Erlotinib, Brivanib, Everolimus, Rapamycin, Linifanib etc. [28]–[30]. These drugs mostly target pathways in the Signal Transduction category, and few in other categories such as Cell Communication, Cell Growth and Death, Cancer, Immune system, and Signaling Molecules and Interaction. Here we study how and to what extent the newest emerging drugs may affect the connectivity of the targeted pathways. Targeting multiple nodes is a common way to measure network robustness and to estimate a drug’s effect on the network connectivity [31],[32]. Although each drug affects its targeted genes differently, with different degrees of impact on their pathways, it is essential to estimate the potential effect on the global network scale, where each drug is assumed to equally affect its targeted pathways. Thus, at this point, we make a simple assumption that drugs with the same targeted pathways have the same effect. From the 18 drugs that we examined (Additional file 8), Sorafenib targeted the most pathways (8 pathways). Therefore, we describe the impact of Sorafenib in detail (and some other drugs in Figure 4), while the results of all drugs can be found in Additional file 8.

We first examined the entire pathway network of cancer type A and modeled the effect of targeting combinations of pathways simultaneously, by deleting nodes from the network (shown in blue, Figure 4). Specifically, we studied the 8 top hubs (5% of all nodes). Although they engage in crosstalk with 91% of all pathways in the network, the connectivity of these 8 top hubs includes 22% of the original network edges. When excluding these 8 hub edges and examining the remaining pathways in the network, we found only a single node that was excluded (in addition to those 8 hubs). Hence, administering drugs that affect only the top hubs would not necessarily cause dramatic changes. Here, we only demonstrate the connectivity of the network with relation to pathways that can be completely targeted by administering different drugs.

### Sorafenib

Sorafenib targets 8 pathways including MAPK, ErbB, Cytokine-cytokine receptor interaction, Chemokine, mTOR, and Natural killer-cell-mediated cytotoxicity (KEGG: D08524). These pathways are correlated with 82% of all pathways in our pathway network, constituting 15% of all interactions (shown in red, Figure 4). However, when deleting Sorafenib’s pathways from the original pathway network of cancer A, most top hubs remain as in the original network, except the following pathways that dramatically lost their high connectivity: Regulation of Actin Cytoskeleton, Leukocyte Transendothelial, Adherens Junction Migration, Focal Adhesion, and ECM-Receptor Interaction. The new hubs, after eliminating Sorafenib’s pathways are Metabolic Pathways, Phagosome, Endocytosis, Axon Guidance, Long-Term Depression, FC Gamma R-Mediated Phagocytosis, Osteoclast Differentiation, Cell Adhesion Molecules (CAMS), and Chemokine signaling pathway. Note that the secondary neighbors of Sorafenib’s pathways (and practically from all drugs) include approximately the entire network, which demonstrates the compact structure of the original network. Still, all the drugs produced limited changes in global connectivity mainly due to the high number of low-ordered circles that tightly connect them. We scanned the 3-node circles that include Sorafenib’s pathways, and found that the three most observed category circles are: [Signal Transduction, Infectious Diseases, Cancers], [Signal Transduction, Immune System, Cancers], and [Signal Transduction, Signaling Molecules and Interaction, Immune System]. Thus, there is a need to understand the crosstalk between Sorafenib’s pathways and the Immune System, Infectious Diseases, and Signaling Molecules and Interaction pathways.

### Immune system

Throughout our analysis of redundancy mechanisms, the pathways of the immune system were found to play an important role in the aggressive HCC cancer type (type A). There are two hubs_gene_ that function as part of the immune system pathways. Moreover, current targeted therapies sometimes target immune system pathways. For instance, both Sorafenib and Lexatumumab target the ‘Natural killer cell-mediated cytotoxicity’ pathway. In addition, all other HCC drugs were shown to crosstalk with immune system pathways in that pathway network. Furthermore, we found that most of the pathways that are included in 3-node circles crosstalk with the pathways of the Immune System.

We further examined the effect of changes in the Immune System’s pathways on network connectivity. We compared the connectivity of 4, 6, or 9 Immune pathways vs. the connectivity of targeted pathway therapies with the same number of pathways (shown in green, Figure 4). The Immune pathways that we examined were Leukocyte Transendothelial Migration, Fc Gamma R-mMediated Phagocytosis, Chemokine signaling pathway, Hematopoietic Cell Lineage, Antigen Processing and Presentation, T Cell Receptor signaling pathway, NOD-Like Receptor signaling pathway, Fc Epsilon RI signaling pathway, and B Cell Receptor signaling pathway (in this order, based on their network connectivity). We found that by comparing the connectivity effects of administering the drugs trastuzumab, bevacizumab, and lexatumumab vs. targeting only the first 4 Immune pathways, trastuzumab and the Immune pathways showed approximately the same high level of first neighbor connectivity compared to those drugs, but the Immune pathways participate in more 3-node circles (15%, 11% and 9% vs. 19% respectively, Additional file 8). For drugs that target 6–7 pathways, we compared the drugs erlotinib, gefitinib, and linifanib with the first 6 Immune pathways, and found that the first 6 Immune pathways have the highest first neighbor connectivity, and highest number of 3-node circles (14%, and 26% respectively). Lastly, we compared the first 8 hubs, sorafenib’s pathways, and the first 8 Immune System most connected pathways. Targeting the hubs produced the most effective results, following by the Immune pathways and then sorafenib. For example, the percentage of 3-node circles included in the 8 hubs, the 8 pathways of Immune System, and the 8 pathways targeted by sorafenib are 37%, 31% and 22% respectively (Additional file 8).

## Discussion and conclusions

The mechanism of redundancy in biology has been studied for decades [33]. Many reports have shown that cancer cells can exploit redundancies in pathways, feedback loops, and crosstalk in order to survive despite the administration of specific drug treatments [34]. As a result, many studies have focused on revealing pathway crosstalk and suggesting methods to estimate the impact of drugs [35]. Cancer can be described as a disease resulting from abnormal intra- and inter-cellular communications. It includes abnormal levels of expression of known pathway crosstalk, but also initiates new cancer pathway crosstalk to deal with unfamiliar stressful conditions such as immune response, hypoxia, toxic drugs, and even metastasis. The resulting network of these abnormal pathway crosstalks is the outcome of all the communication of cancer cells with their environment. Thus the expression of these extracted cancer cells should include evidence of these prior cellular communications. The ability to predict cancer pathway crosstalk would contribute to our understanding of the cellular functions of cancer cells and help predict the response of those cells to various treatments. We introduce here a computational methodology to analyze gene expression data from hepatocellular carcinomas of varying responsiveness and non-tumor hepatocytes, and offer treatment strategies that are designed to overcome redundancy and resistance mechanisms. We discuss the impact of three mechanisms of redundancy, and demonstrate in detail their effect on the connectivity of the crosstalk between the MAPK signaling pathway and the Focal Adhesion pathway. The MAPK pathway is one of the pathways most targeted by current HCC treatments, and Focal Adhesion is one of the most communicated pathways in the cancer type A network; thus, the crosstalk between them is a unique and important communication that should be examined thoroughly.

First, we estimated the effect of redundant genes by separately deleting or perturbing genes that participate in the MAPK pathway: FGFR3, FGFR4, FLNA, and AKT3. The first two genes are part of the MAPK pathway, but the second two genes are also involved in Focal Adhesion. This crosstalk is still statistically significant (*p*-value < 0.05) when excluding FGFR3, FGFR4, and AKT3, although there is some variation in their pathway weights. Second, we explore the redundant short paths between the MAPK and Focal Adhesion pathways through an intermediate pathway, by finding all 3-node circles (also termed *triangles*). We found 63 intermediate pathways that engage in crosstalk with both pathways. Therefore, targeting one or more of those 63 pathways would probably not disrupt the crosstalk between the MAPK and Focal Adhesion pathways, which would be considered an undirected approach. Third, we examined if those 3-node circles are part of the same biological system. In other words, are they redundant circles? Intermediate pathways can be grouped together based on their biological properties (i.e., pathway category). For instance, the Leukocyte Transendothelial Migration, Toll-Like Receptor signaling pathway, Hematopoietic Cell Lineage, and Fc Gamma R-Mediated Phagocytosis pathways are all part of the Immune System category. Thus, the 63 circles can be grouped and the crosstalk frequency of each biological system with MAPK and Focal Adhesion pathways can be estimated. We were able to group 9 circles related to Infectious Diseases, 8 related to the Immune System, 5 to Signal Transduction, 5 to Cancers, 4 to Translation, etc. (Figure 3).

### Network structure reveals weak spots in the cancer network

There are many ways to study the vulnerability and robustness of a network, but all are dependent on the network structure. In this work we demonstrate how the global and local network features can guide us to find better targets to treat HCC. Among other features, we discussed the hubs in both networks. We found many confirmations that the hubs_gene_ are clinically and experimentally observed to be important to the development and treatment of HCC, and to solid tumors in general. For instance, CD47 functions as part of the ECM-receptor interaction pathway. CD47 is also a ligand for SIRPα, a protein expressed on the surface of macrophages and dendritic cells. Recently, Weiskopf et al. [36],[37] described an antibody-mediated tumor immunotherapy that overcomes resistance. Their analysis of patient tumors and matched adjacent normal tissues suggests that all human solid tumor cells require CD47 overexpression to suppress phagocytic innate immune function, and suggests CD47 as a validated target for cancer therapies. A second example is IQGAP1, a member of the IQGAP family of scaffold proteins, and a key mediator of cell adhesive and cytoskeletal rearrangements. IQGAP1 binds to many cancer-related proteins, such as Cdc42, Rac1, E-cadherin, beta-catenin, calmodulin and members of the MAPK pathway [38],[39]. IQGAP1 overexpression has been observed in numerous kinds of tumors. It affects the development of HCC by regulating many important signaling pathways, such as cell proliferation, motility, and invasion. Future developments related to IQGAPs may reveal new therapeutic targets [40],[41].

Targeted therapies have been developed using several promising drugs for advanced HCC, including sorafenib, sunitinib, brivanib, cetuximab, everolimus, erlotinib, and lapatinib [42]–[44]. Sorafenib is a small molecule that inhibits tumor cell proliferation and angiogenesis and increases the rate of apoptosis in a range of tumor models. Ongoing studies and trials are evaluating the efficacy and tolerability of combining/sequencing Sorafenib with other targeted agents that inhibit different/parallel pathways in HCC (e.g., erlotinib, sunitinib and brivanib [42]). An open question is whether synergy is more likely to occur by combining drugs that share the same pathways at high doses or those that affect highly connected pathways at lower doses.

Choosing drugs for therapy is a complex task. Researchers often choose a specific element to target (e.g., VEGFR2 tyrosine kinase inhibitor) using statistical analysis of gene expression, or the target’s ability to affect cell fate (i.e., does the target act as an upstream hub?). However, many cancer drugs fail or underperform due to redundancies in their target’s pathways or the existence of alternative pathways. Efficiently targeting pathways is problematic, because it is unclear whether we should identify pathway targets by level of expression or by their location in the pathway (e.g., upstream elements). Determining a pathway network-based redundancy consisting of genes that are over-expressed in poor prognosis HCCs could help to isolate targets that when inhibited would disrupt or destroy the cancer network and hopefully increase the probability of cell death.

We estimated the relationship between the network connectivity and its hubs, and found that inhibiting the first 8 hubs had a substantial effect on the remaining sub-network, mainly due to the compact structure of the network where the hubs are also part of many regulated small circles. Also, we compared the results from Sorafenib’s 8 targeted pathways, as compared to the 8 highest connected Immune System pathways. The Immune System pathways were found to be part of most of the 3-node circles. The results clearly showed that the Immune System pathways closely regulate most pathways, and therefore dramatically change the network connectivity when they are targeted, more than all currently employed HCC targeted drugs. One prediction from these studies is that sorafenib combined with a drug that inhibits Immune System pathways as compared to combination with another targeted drug, may lead to better treatment outcomes. Many of the immune system genes whose expression is studied here are likely to be derived from lymphocytes, neutrophils and macrophages that have infiltrated the HCC tumors. Although expression of these genes in the tumors themselves cannot be ruled out, these results suggest that there is crosstalk among HCC gene pathways and immune system cell pathways, and that targeting the immune cell pathways can affect treatment outcomes for individuals with HCC.

The novelty of this study is not defining new network features, but creating a multilayered network that includes genes, pathways, and pathway families using gene expression data. By comparing the different networks (for different cancer types and non-tumorous tissue), and by evaluating the redundancy on different levels, we can estimate the effect of each element, in a multilayered network, on the biological phenotype.

### Future perspectives

In this initial analysis, we have demonstrated changes at different network levels by completely excluding the targeted genes, and have examined the impact on the network connectivity with relation to different drug targets. A future approach to improve prediction of drug effects on the pathway network would involve changing the initial gene expression after inhibiting several main pathways (and consequently the two initial gene and pathway networks) and only then comparing the changes propagated throughout the network. This process would require data gathered before and after treatment, ideally in cancer studies in vivo, or in ex vivo systems that mimic in vivo physiology. Moreover, our method could be further expanded to study the pathway network based on data from a single patient, so intratumoral heterogeneity and individual variation would be considered. In addition, the redundant mechanisms of classical multidrug resistance could be discussed using our approach.

In this paper we discuss the importance of ‘node degree’ in our networks, in addition to other features. But, there is a complementary network feature to ‘hubs’, i.e., bottlenecks. Bottlenecks can be defined as nodes with a high “betweenness centrality” [45]. Bottlenecks are, indeed, key connector nodes with properties that relate to the function and dynamics in interaction networks. However, as Goh and his colleagues reported, in several interaction networks that they examined, the betweenness of a node is correlated to its degree [46]. Therefore, it is not clear whether node bottlenecks are important due to their high betweenness or high degree values. Determining this would require detailed information about the differences between the bottlenecks and hubs in co-expression networks vs. interaction networks. These aims will be the subject of future work.

## Methods

### Differentially-expressed genes

In order to specifically address the gene and pathway communications in HCC, we use the Differentially-Expressed Genes (DEGs) profile of HCC as our first step. Hoshida and colleagues defined and validated three gene expression signatures of common molecular subclasses of HCC. They presented a meta-analysis of gene expression profiles in data sets from 9 independent patient cohorts on different microarray platforms [7]. A total of 603 patients from Western and Eastern countries with HBV and HCV were analyzed. They observed three robust HCC subclasses (termed S1, S2, and S3) that were correlated with clinical parameters. The S_1_ and S_2_-signatures reflect more aggressive tumors. The data are available at Gene Expression Omnibus (GEO), accession nos. GPL1528, GPL2094, GPL80, GPL257, GPL91, GPL96, GPL570 and GPL5474. The S_1_-signature includes 226 genes, the S_2_-signature 115 genes, and the S_3_-signature 261 genes. For further details about the datasets used for subclass definition and validation see Supplementary Table one in Hoshida et al. [7]. For clinical phenotypes associated with HCC subclasses see Table one in that paper [7], and for clinical demographics in 9 HCC datasets see Supplementary Table two, also in Hoshida et al. [7]). Our initial list of differentially expressed genes is based on these three signatures, listed in full here in Additional file 1.

### Gene expression profiling data

Our second step was to choose the data to construct the co-expression network. We used publicly available gene expression data sets from a study by Thorgeirsson and colleagues [22]. These data sets were also used by Hoshida and colleagues to identify differentially-expressed genes. The data are available at GEO, accession nos. GSE1898 and GPL1528. Ninety-one HCC tissues and 60 matched non-tumor surrounding liver tissues were obtained from 90 patients undergoing partial hepatectomy as treatment for HCC. Tumor specimens originated from patients with HBV and HCV. These samples were classified by Lee et al. into two groups of cancer based on overall survival, where group A demonstrated poor overall survival (correlating with signatures S_1_ and S_2_) and group B demonstrated good overall survival (correlating with signatures S_3_). For more details about clinical and pathological features of HCC patients see Table one in Lee *et al.*[22]. We normalized the data of Thorgeirsson and colleagues using the quantile-normalization method.

### Gene co-expression network

The third step is to define the relationship between every pair of genes (i.e., edge) and to create a network. The gene network is structured as the ‘approved’ statistical correlations between every pair of genes, where genes are *nodes* and correlations are *edges*. The number of nodes does not necessarily equal the entire gene expression list of genes; here it is based on their statistical correlations. We filtered out genes with absolute expression levels as within the lowest 10 percent of the data set and with variance (across samples) in the lowest 10 percent. We computed pairwise correlations for all possible pairs of genes from our expression data using Pearson statistical correlation, and define a significant gene edge by: (1) any correlation value with (2) False Discovery Rate (FDR) *adjusted-p-values* < 0.05. A weighted gene network based Pearson correlation (with no threshold) and its higher-correlated sub-network (|correlation| ≥ 0.5) are constructed separately for each HCC type. The correlation value is known to be dependent on the number of samples. Therefore, the number of samples for all networks from the same type was fixed based on each cancer group.

At the end of this process, there is a single gene network for each data group (cancer type A and cancer type B). Each cancer network was studied in two forms: every gene edge satisfies the *adjusted-p-values* < 0.05 with no thresholds, or *adjusted-p-values* < 0.05 with gene |correlation| ≥ 0.5.

### Randomization, permutation test, and statistical significance of a network

To address the question of phenotype specificity, we compared the cancer network to the random networks from the same cancer type, where the random network combines expression data from the specific cancer group and the matched non-tumor group, using the same initial gene list (signatures S_1_ + S_2_ for comparison with cancer type A, or signature S_3_ for cancer type B). We used the permutation re-sampling method [47],[48] of the original data to model the null distribution. We combined the raw gene-expression data from the cancer group and its matched non-tumor group, so the total numbers of samples were the same as the original. Then we randomized the labels of the samples (cancer and non-cancer) while fixing the number of samples to ‘m’, and calculated the ‘approved’ network. This procedure was repeated 150 times to create 150 *random networks per cancer type* in order to calculate the p-value. Using this method, we determined the statistical significance of each network characteristic/feature, and the significance of each pathway edge. See example listed in Additional file 3.

### Network characteristics

The topological features of a network can be described by several statistical metrics [4],[49],[50]. These statistical metrics can help to reveal the biological relevance of the network. Several network characteristics were used in the text (also see Additional files 2, 3, 4 and 5): *Node degree* (*k*) is the number of edges by which a node connects to other nodes. The fraction of nodes in the network with degree *k* is called *degree distribution* (*p(k)*). *Hubs* are nodes with high degree nodes. The *edge density* of a network, also called connectivity, is the proportion of edges that exist relative to the number of potential full connections of a network. *The local clustering coefficient* of a node measures how close its neighbors are to being a complete graph, and gives the proportion of a node’s neighbors that are connected. The clustering coefficient for the whole network can be calculated as *the average of the local clustering coefficients* of all nodes [49],[50]. A *connected component* is a set of connected nodes in the network (sub-network). The largest connected component in a network is called the *giant connected component*.

### Pathway co-expression network

We generalized the gene network to a pathway network, with each gene interaction translated to all possible pairs of pathways, and estimated their likelihood. The pathway network is composed of pathways as nodes and correlations as edges. Each gene correlation was translated to a pathway correlation using the final gene co-expression network and the KEGG pathways database (Kyoto Encyclopedia of Genes and Genomes, www.genome.jp/kegg/). To address the question of its specialty to a specific phenotype, we compared the pathway network to 150 random pathway networks, and using a permutation test we calculated the *p*-value of each pathway edge. All pathway edges with *p*-value < 0.05 were assumed to be significant and the resulting pathway network was reported in the main text of our paper (see Randomization and Statistical Significance).

### Database and computational programs

All data concerning genes and pathways were downloaded from the KEGG database (Kyoto Encyclopedia of Genes and Genomes) [51]. For the network analysis we used the computing program Matlab, whereas all network feature procedures can be found in the Complex Networks Package for MatLab (Version 1.6; Muchnik, L.) and in [52]. All network visualizations were performed using the software Cytoscape (www.cytoscape.org/).

### Availability of supporting data

The data sets supporting the results of this article are available in the Gene Expression Omnibus (GEO) repository, accession nos. GPL1528, GPL2094, GPL80, GPL257, GPL91, GPL96, GPL570 and GPL5474. These can be found at http://www.ncbi.nlm.nih.gov/gds.

## Abbreviations

DEG: Differentially-expressed gene

GCN: Gene co-expression network

HCC: Hepatocellular carcinoma

## Competing interests

The authors declare that they have no competing interests.

## Authors’ contributions

OL and MG conceived the project. Experiments were designed and performed by OL. Data were analyzed by OL and JS. All the authors contributed to writing the paper. All authors read and approved the final manuscript.

## Additional files

## Supplementary Material

Additional file 1 Gene and pathway annotation. Click here for file

Additional file 2 Properties of Gene Co-expression Network. Click here for file

Additional file 3 Gene Network properties of Random vs. Cancer Type A. Click here for file

Additional file 4 Gene Network properties of Random vs. Cancer Type B. Click here for file

Additional file 5 Properties of the Pathway Network. Click here for file

Additional file 6 3-node circle categories. Click here for file

Additional file 7 Node degree of gene and pathway networks. Click here for file

Additional file 8 Drug targets. Click here for file
