# Supplementary figures and images for: Network features suggest new hepatocellular carcinoma treatment strategies
Source: BMC Syst Biol. 2014 Jul 29;8:88. doi: 10.1186/s12918-014-0088-0 (PMC4236726; doi:10.1186/s12918-014-0088-0)

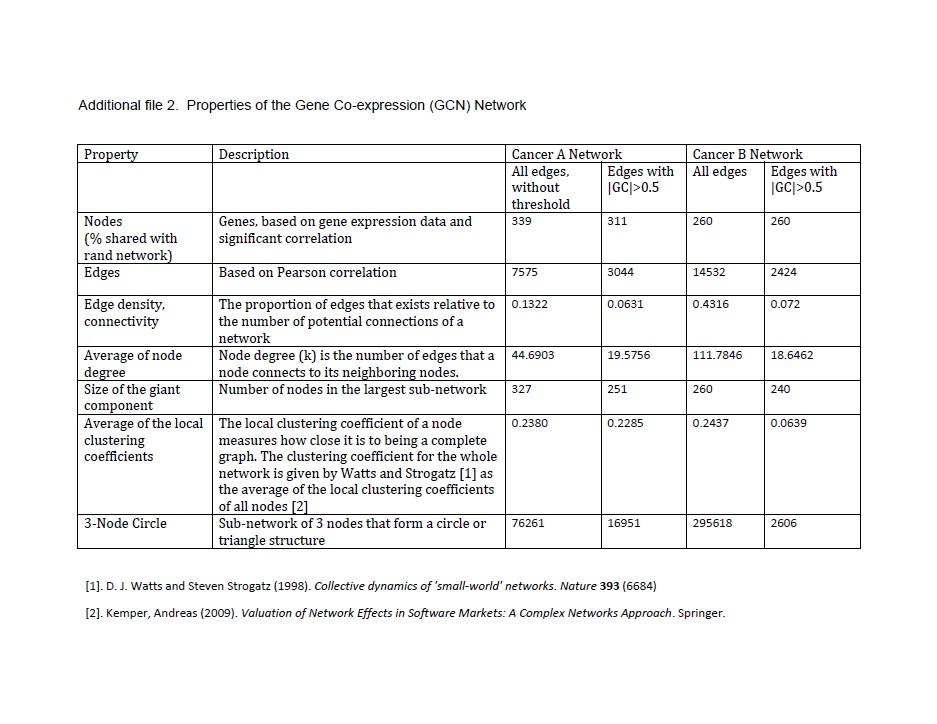

Supplement: Additional file 2 — Properties of Gene Co-expression Network. [file s12918-014-0088-0-S2.jpg]

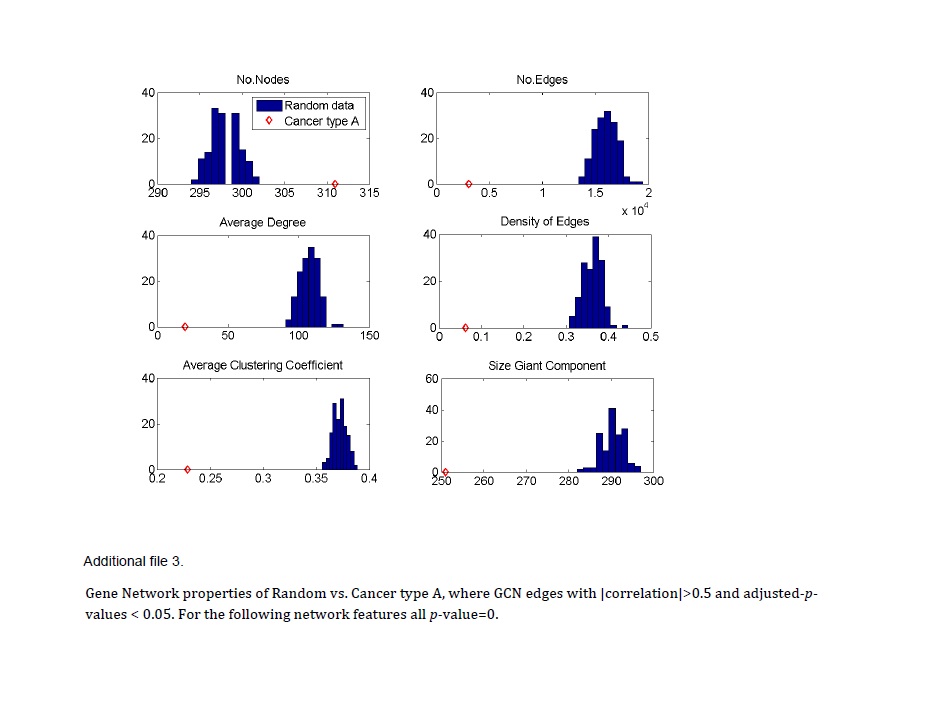

Supplement: Additional file 3 — Gene Network properties of Random vs. Cancer Type A. [file s12918-014-0088-0-S3.jpg]

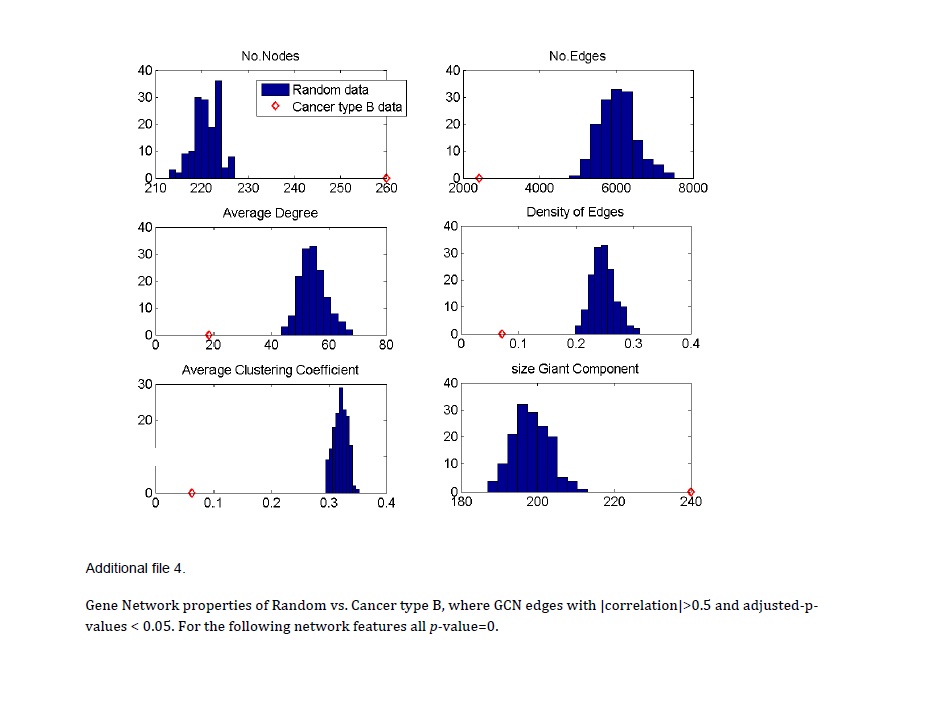

Supplement: Additional file 4 — Gene Network properties of Random vs. Cancer Type B. [file s12918-014-0088-0-S4.jpg]
