# Supplementary material for: Network features suggest new hepatocellular carcinoma treatment strategies
Source: BMC Syst Biol. 2014 Jul 29;8:88. doi: 10.1186/s12918-014-0088-0 (PMC4236726; doi:10.1186/s12918-014-0088-0)
Supplement: Additional file 5 — Properties of the Pathway Network. [file s12918-014-0088-0-S5.pdf]

Table S3 . Properties of the Pathway Network

Property	Cancer A Network:		Cancer B Network	
Pathway edges with:	|WP|>0.01	|GC|>0.5, and WP>0.01	|WP|>0.01	|GC|>0.5, and |WP|>0.01
Nodes	168	154	176	176
Edges	4768	3468	8526	5814
Edge density, connectivity	0.3399	0.2944	0.5536	0.3775
Average of node degree	56.7619	45.039	96.8863	66.0682
Number of connected components	6	21	2	2
Size of the giant component	161	133	174	175
Average of the local clustering coefficients	0.3125	0.4104	0.3421	0.3221
No. Circles with 3 Nodes	44439	42390	160453	84557

WP= Weight of Pathway Edge, GC=Gene Correlation
